# Detecting Polystyrene Nanoparticles in Environmental Samples: A Comprehensive Quantitative Approach Based on TD-PTR-MS and Multivariate Standard Addition

**DOI:** 10.1021/acsestwater.5c00054

**Published:** 2025-08-01

**Authors:** Nematollah Omidikia, Helge Niemann, Hanne Ødegaard Notø, Rupert Holzinger

**Affiliations:** † Department of Marine Microbiology and Biogeochemistry (MMB), Royal Netherlands Institute of Sea Research (NIOZ), ‘t Horntje 1797 SZ, The Netherlands; ‡ Institute for Marine and Atmospheric Research, IMAU, 8125 Utrecht University, Utrecht 3584 CC, The Netherlands; § Department of Earth Sciences, Faculty of Geosciences, Utrecht University, Utrecht, 3584 CB, The Netherlands

**Keywords:** nanoplastics, quantitative analysis, thermal desorption, non-negative matrix factorization, matrix effect, curve resolution

## Abstract

Submicrometer-sized plastic particles (nanoplastic; NP) have been detected in a large variety of different ecosystems. They occur in small quantities within a complex organic matrix comprising a plethora of compounds. A robust quantification of the NP concentration thus requires the development of a comprehensive analytical workflow to handle potential interferents. Thermal desorption–proton-transfer reaction–mass spectrometry (TD-PTR-MS) creates the necessary chemical selectivity to distinguish NP signals from the organic matrix. Nevertheless, the recorded raw mass spectra are too complex for direct interpretation, and further signal clustering/scoring is required for a more in-depth analysis. Here, we resolved this problem in a novel workflow, which combines non-negative matrix factorization (NMF) and multivariate standard addition (MSA). This allows us to mathematically separate the NP’s signature from the mixture, as showcased for polystyrene nanoparticles. The method produces an unequivocal and matrix-corrected NP fingerprint for identification and quantification. MSA and NMF enabled us to quantify polystyrene NP in different environmental samples in the lower nanogram range. The mass concentration of polystyrene NP in Waal River water sampled close to Nijmegen, the Netherlands, was 4.7 ± 0.65 ng/mL and 39 ± 0.70 ng/g in sand samples from the river’s shore. A sand sample from a local playground in Nijmegen exhibited a higher concentration of 129 ± 1.1 ng/g.

## Graphical abstract

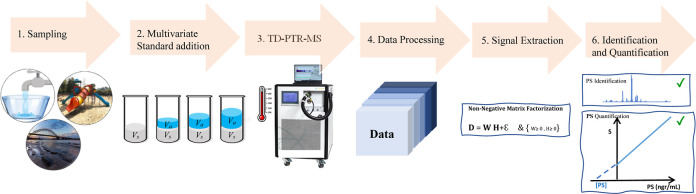

## 1 Introduction

Plastics have been produced abundantly and utilized for various purposes due to their relative affordability and ease of manufacturing.^1,2^ Regrettably, only a small fraction of plastic waste is recycled, while the vast majority ends up in landfills and/or the environment.^3–5^ Primary plastic waste typically comprises fragments >5 mm (macroplastics), but various physicochemical factors contribute to the gradual reduction of the plastic fragment size from macro to micro (1 μm–5 mm) and nanoparticles (≤1 μm).^6–10^ Nanoplastics (NPs) are more easily transported than larger particles, making their presence notable even in high-altitude mountains, remote regions, and the ocean.^11–15^ Independent of the polymer type, NPs in water are quasi-dissolved because their dispersion is not governed by buoyancy properties but more dominantly controlled by the collision of nanoplastics with water molecules and Brownian motion.^6,7^

NP contamination poses potential threats to both ecosystems and health.^16^ When exposed to NPs, several potential risks, including mitochondrial impairment, cytomembrane destruction, cell mortality, cytotoxicity, inflammation, oxidative stress, and abnormal metabolism, have been reported.^17–19^ More importantly, the health effects of plastics become more pronounced with higher concentrations and smaller particle sizes.^20,21^ Finally, the ecotoxicological effects of NPs as well as their implications for human health have been studied.^22–24^

The first response to such a threat is thus to better understand the distribution of NPs in the environment to assess the severity of the problem.^25^ Consequently, several analytical techniques to measure NPs with spectroscopic^26,27^ and mass-spectrometric schemes^28,29^ have been developed. Pyrolysis gas chromatography–mass spectrometry (Py-GC\MS) is widely employed for micro- and nanoplastic detection.^28,30^ The pyrolysis step is operated at 500–700 °C, and it is followed by separation and detection steps.^31^ Such a high temperature partially degrades the polymers in the sample matrix. Additionally, copyrolysis increases the interaction between different components during thermal analysis, boosting the NP quantification uncertainty.^32^ Nanoplastic loads of ≥50 ng are typically required for a Py-GC\MS analysis. Another thermo-analytical method coupled with mass spectrometry is thermal desorption–proton-transfer reaction–mass spectrometry (TD-PTR-MS), a newly emerging analytical tool for the quantification of nanoplastics in environmental samples.^33,34^

Environmental samples are inherently complex, with NPs constituting only minor traces of the total.^15,25,35^ Therefore, to identify and quantify nanoplastics, it is crucial to differentiate the pure NP signal from signals generated by other compounds in a sample. A TD-PTR-MS method was successfully used by Materic et al.^11,15,33^ for the semiquantitative analysis of NPs with a detection limit on the order of 1 nanogram. Their method features high sensitivity and a low detection limit and enables high sample throughput analysis without the necessity for preconcentration.^11,33^ However, this method requires signal scoring/fingerprinting and specific thresholding to detect the polymer backbone, which introduces some levels of ambiguity. Moreover, the plastic fingerprint does not account for instrument variability over time (e.g., drifts in MS properties) and different technical specifications between instruments, which introduces additional inaccuracy. Though these issues can be minimized, it remains challenging to eliminate them. Proper interpretation of the measurements requires highly specialized and experienced personnel. Nevertheless, the current TD-PTR-MS method has been a breakthrough; however, its wider application is limited by the complexity of data interpretation, and the reliability of its quantitative analysis remains uncertain.

In this contribution, we address these issues by separating the minor plastic signal from the complex sample matrix using non-negative matrix factorization (NMF) to improve data processing and extract NP signals. We further combine NMF with a multivariate standard addition (MSA) protocol, enabling us to (i) unequivocally extract the NP signature and (ii) quantitatively analyze nanoplastics in environmental samples with complex matrices. The combination of a MSA protocol with NMF provides a novel and robust TD-PTR-MS approach that is fully quantitative. We tested the developed workflow on several synthetic and environmental samples.

## 2 Materials and Methods

### 2.1 Sampling

We analyzed three types of samples: (i) tap water samples were collected from the laboratory at Utrecht University (July 21, 2023 at 8:00 am). For this, the water was filled into a 10 mL syringe (Terumo syringe; Terumo Corporation, Tokyo, Japan) directly from the tap (after rinsing the syringe 3 times with the same tap water). The water was then filtered through a PTFE syringe filter (0.2 μm pore size; GE Healthcare, USA) to exclude potentially larger particles in the subsequent analysis. The first few mL of filtered water were discarded to prevent potential contamination released from the filters. The water was then collected in prebaked (330 °C for at least 48 h) 10 mL chromatography glass vials (VWR, Germany) in a laminar flow hood. The vials were immediately closed with 1.5 mm screw caps (VWR International, Darmstadt, Germany) (the lid septum was priorly rinsed with the filtered water). (ii) River water and riverbank sand samples were collected in Nijmegen, the Netherlands, from the Waal River coast (51°51′02.0″ N, 5°51′34.9″ E; 23 July 20, 2023 at 13:00). For this, the water was collected from the shore by immersing prebaked 10 mL chromatography glass vials, which were immediately closed with caps (priorly rinsed in the river water). In a laminar flow hood, the river water sample was then filtered through PTFE syringe filters and collected in 10 mL chromatography glass vials (see above for syringe, filter, and vial specifications). From the same location where we collected water, we also collected sand from the Waal’s shore in 10 mL chromatography vials using a prebaked stainless steel spatula. (iii) Finally, a sand sample was collected from a playground (at random spots inside the playground) close to the city center in a very populated area in Nijmegen, the Netherlands (51°50′44.8″ N, 5°51′30.8″ E; 22 July 2023 at 17:00). Using a stainless-steel spatula, the sand was collected in 10 mL chromatography glass vials.

### 2.2 Preparation of Samples, Process Blanks, and Standards

For clarity and consistency, all experiments were conducted using prebaked chromatographic vials (heated to 350 °C for at least 48 h) to prevent nanoparticle contamination. Additionally, all sample preparation and laboratory work were carried out under a laminar flow hood.

For polystyrene (PS) NP extraction from sand, a sand-water extraction was performed without any pH adjustment.^36^ Ultrapure water (HPLC grade) was added to the sand with a ratio of 1:4 (1 g of sand: 4 mL of HPLC water). The mixture of sand/water was well mixed (vortexed) for 15 min and then sonicated for 6 h at 60 °C. Finally, the supernatant fluid was filtered through a PTFE syringe filter (see specifications above).

Process blanks were prepared using similar amounts of Milli-Q water to rinse the same containers and surfaces as used for sampling and sample preparation, including laboratory materials such as syringes, pipet tips, volumetric flasks, and filters. At the sampling site, field blanks were obtained by filling HPLC water in chromatography vials to mimic river water sampling. We also immersed a prebaked spatula in a chromatography vial filled with HPLC water. This was done to assess potential contamination in the sampling procedure. Subsequently, these blanks underwent the same processing steps as the actual samples, enabling the evaluation of potential sources of contamination during the sampling and analysis workflow.

PS standards in the range of 1–100 ng mL^–1^ were prepared by diluting polystyrene spheres (300 nm diameter, PS-ST-1.0, Microparticles GmbH, Berlin, Germany) in HPLC water (VWR, filtered through 0.2 μm, CAS 7732-18-5). Finally, a low-pressure evaporation/sublimation unit was used to remove water from samples as described in the previous series of work based on TD-PTR-MS.^15,37^

### 2.3 TD-PTR-MS Measurement and Semiquantitative Analysis

The TD-PTR-MS protocol and parameters are based on previously optimized conditions for NP detection.^33^ TD temperature was ramped from 35 to 350 °C at a rate of 40 °C/min and then held constant at 350 °C for 5 min to ensure desorption of all NP content from the vial. Further evidence supporting the adequacy of the TD temperature program is provided in the Supporting Information. The thermogram of the styrene ion and the TGA analysis of PSNPs are summarized in Figures S2 and S3, respectively. The PTR-TOF-MS (PTR4000 Ionicon Analytik, AT, equipped with a hexapole lens and ion funnel) measured the outflow in real time (1 Hz resolution). We used the following operation parameters of PTR-TOF-MS: inlet temperature = 180 °C, drift temperature = 120 °C, drift tube pressure = 2.9 mbar, drift tube voltage = 480 V, and a reduced electric field strength (E/N) of 105 Td (1 Td = 10^–17^ V cm^–2^).

Raw high-resolution mass spectra from PTR-TOF-MS were processed using the custom-made software package PTRwid.^38^ The PTR-MS signals recorded during the TD operation were integrated within the temperature window of 150 to 350 °C, leading to an averaged mass spectra per sample. The purpose of this truncation-integration is 2-fold. (i) Chemical selectivity is created by removing volatile organic compounds and possible monomeric contaminations. (ii) Integrating the PTR-MS signal over the temperature accumulates signals of the nanoparticles’ degradation products, resulting in a representative signal for each sample. For example, evaporation of styrene monomers is fully completed at *T* < 145 °C, so that styrene monomers are excluded from the analysis. Scheme 1 presents a graphical overview of the collected data set from a series of samples, followed by merging/integration. TD-PTR-MS generates a matrix per sample with the dimensions of scanned temperature by *m*/*z* channels. In the case of multiple samples, a data cube is created. This third-order tensor contains the thermograms of the chemicals (i.e., NPs) and information about the fragmentation and degradation of chemicals.^33^ Integration within the temperature mode builds the final data matrix for further processing.

**Scheme 1:**
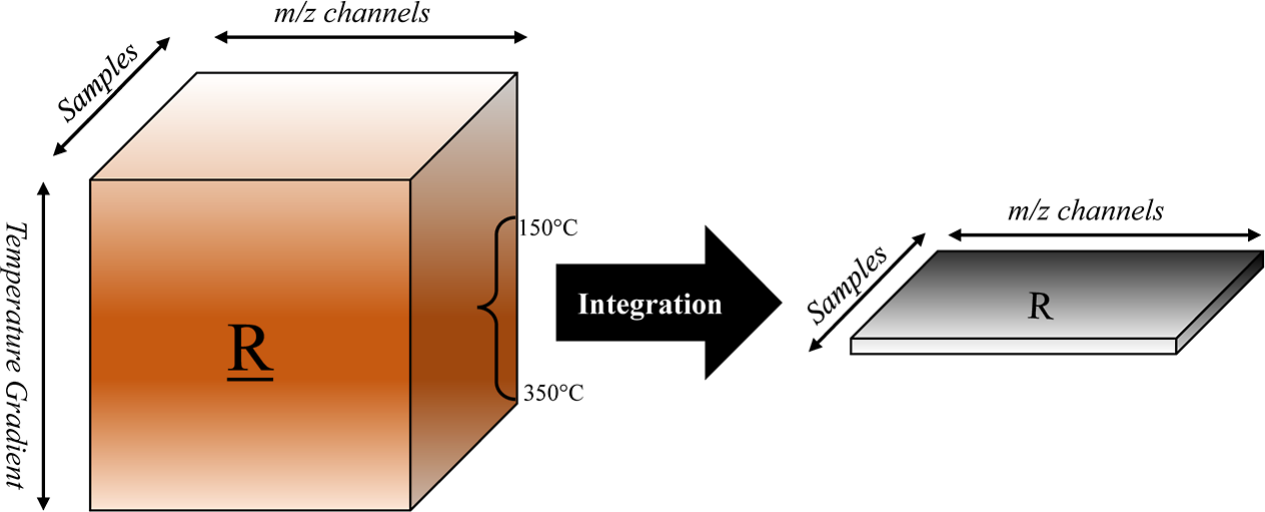
Schematic Overview of the Data Set Generated by TD-PTR-MS for Different Samples

One of the unique features of the PTR-MS is the possibility of semiquantitative analysis for the desired analyte concentration according to^33,39^

1 $$\left[M\right]=\frac{1}{kt}\times \frac{\left[MH^{+}\right]}{\left[H_{3}O^{+}\right]}\times \frac{Tr\left(m_{H_{3}O^{+}}\right)}{Tr\left(m_{MH^{+}}\right)}$$

In eq 1, [M] is the analyte concentration, which can be converted to a mole fraction considering the pressure and temperature in the drift tube, where *k* is the reaction rate coefficient, *t* is the residence time of the primary ions in the drift tube, and [MH^+^] and [H_3_O^+^] indicate the ion counts representing the protonated analyte and the proton donor, hydronium, respectively. Finally, Tr represents the transmission functions of the hydronium and protonated analyte.^39^ The analyte concentration can be modified to NP concentrations per volume considering the sample load and dilution factor.

### 2.4 Multivariate Standard Addition

Classical external calibration models the linear relationship between concentration and response properly when all chemicals involving the matrix of the samples are equally present in the calibration step.^40^ Consequently, external calibration fails in the presence of unknown interferents. Additionally, several physicochemical factors may violate the linearity of recorded response and matrix effects.^41^ Matrix matching can effectively suppress matrix effects when a blank is available; however, the real challenge is to obtain an acceptable analytical blank because (i) the matrix typically changes from sample to sample and (ii) it is practically impossible to obtain a sample that is free of NPs (i.e., a blank).

To quantify PS nanoparticles in environmental samples, an external calibration curve can be established by using HPLC-grade water in the laboratory. However, in real samples, numerous chemicals contribute to the recorded signal as interferents. Moreover, several factors related to the measurements (e.g., instrument drift) can also impact the signal intensity of the analyte of interest (in this case, PS NPs). Solving both scenarios requires the incorporation of a standard addition method.^40,41^ For this, standards are incrementally added to the test sample containing an unknown quantity of the analyte that must be determined. This approach inherently corrects for matrix effects because the standards are added directly to the sample, making separate matrix matching or an analytical blank unnecessary.^40^ Finally, the concentration of the analyte in the sample is determined by extrapolating to a zero response value.

Figure 1 summarizes the proposed workflow for polystyrene nanoparticle quantification. First, the environmental sample with an unknown amount of PS NPs was subjected to the multivariate standard addition protocol (see Figure 1a). The multivariate standard addition (MSA) process involves dividing an unknown sample into several equal-volume portions. Subsequently, standard solutions of PS NPs, varying in concentration, are spiked into all (but one) subsamples (Figure 1a). Next, the TD-PTR-MS spectra of these samples were recorded and preprocessed using PTRwid software. Then, the raw processed data matrix consists of integrated TD-PTR-MS spectra for the unknown sample (first row of the data) and the titrated sample with standard polystyrene NPs (see Figure 1a,b). The bilinear decomposition of such a mass data set produces two smaller matrices^42^ (see Figure 1c): a concentration matrix, ***C***_***I*** *,* ***n***_, with the information about the concentration of the polystyrene as the analytes, and a factor matrix, ***S***_***I***, ***n***_, with the pure signature of PS.

**Figure 1:**
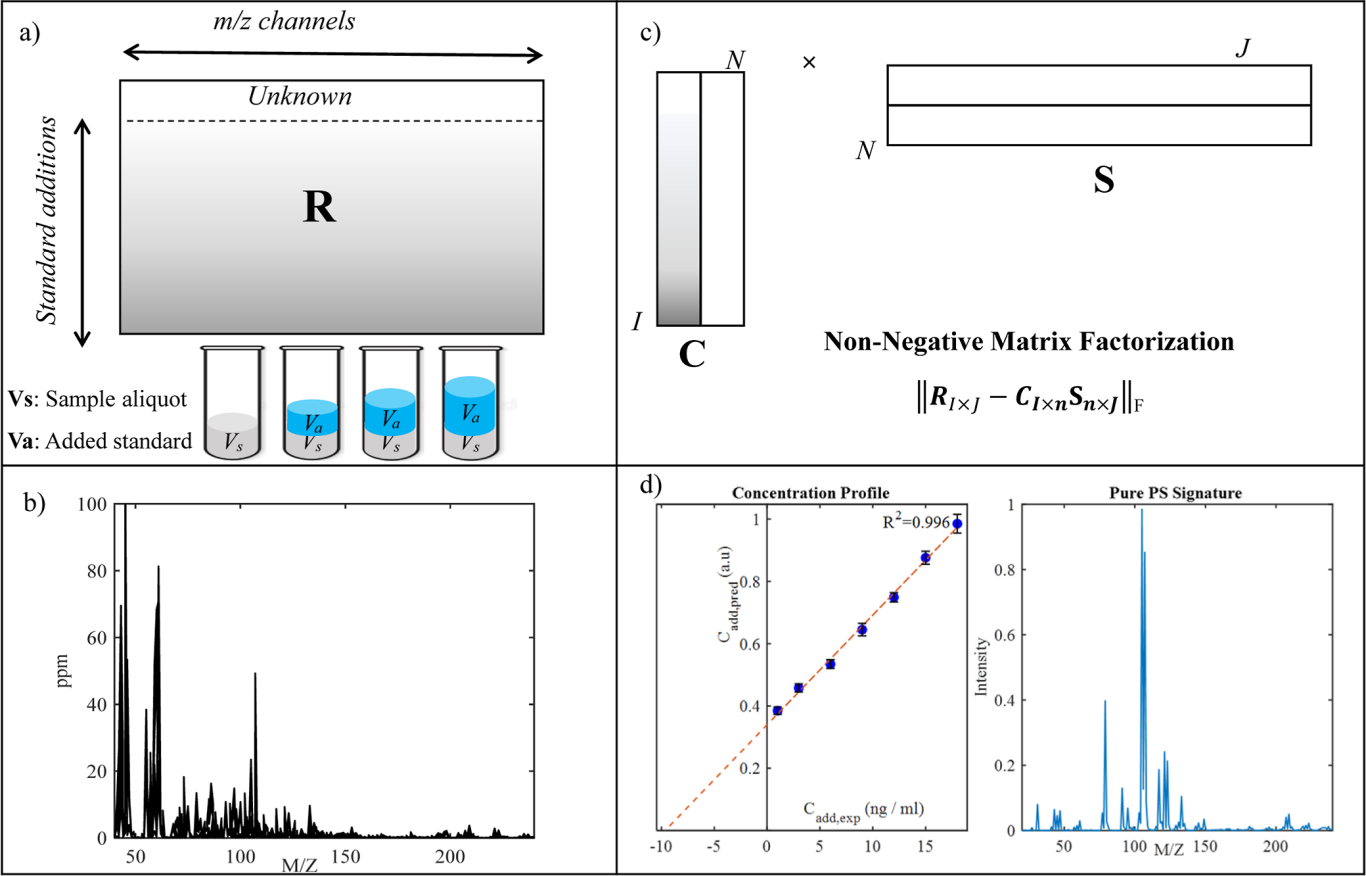
A schematic workflow of the multivariate standard addition method to analyze polystyrene nanoparticles. The unknown sample is spiked with the analyte of interest (PS in our case). For samples from the standard addition protocol, TD-PTR-MS signals were recorded and processed to form a data matrix, ***R***_*I*,*J*_ (a). Panel (b) shows typical data from PTR-MS measurements. Panel (c) illustrates a graphical representation of the bilinear decomposition of the recorded data (***R***_*I*,*J*_) into the matrices of ***C***_***I***, ***n***_ and ***S***_*J*,*n*_ using non-negative matrix factorization. After deconvolution, the component matrices (***C***_***I***, ***n***_ and ***S***_*J*,*n*_) can be subjected to further analysis. The ***C***_***I***, ***n***_ matrix contains concentration information, and ***S***_*J*,*n*_ has pure ion signatures. Panel (d) highlights a standard addition plot, which is spiked PS NP concentration vs NMF-extracted concentration factor (a column in ***C***_***I***, ***n***_). The corresponding row in ***S***_*J*,*n*_ is the pure polystyrene signature. The sample analyzed and presented in the figure is the sand sample from the Waal River. In interpreting sand samples, a dilution factor of 4 should be considered.

The quantitative information can be extracted from ***C***_***I***, ***n***_, which contains the concentration profiles of every analyte (i.e., the evolution of PS NP concentration along the whole addition process). Then, the linear plot of the column of ***C***_***I***, ***n***_ as a function of the added PS-NP concentration allows the same extrapolation process as that in the classical standard addition method (see Figure 1d). The information retrieved by ***S***_***I***, ***n***_ is the pure polystyrene nanoparticle signature, and the corresponding column in ***C***_***I***, ***n***_ is the concentration information (see Figure 1d).

### 2.5 Non-Negative Matrix Factorization

Due to the abundant number of chemicals contributing to the total signal, a selective channel for employing a classical univariate standard addition is hardly available (and/or trustable).^41^ For example, finding a selective *m*/*z* that can only be attributed to a specific plastic type is practically impossible due to the complex sample matrix.^33^ Hence, it is necessary to deconvolute the full mass spectra to recover the contribution of each analyte (here, plastics) to the overall recorded response and benefit from multivariate advantages. The NMF is an appropriate method to mathematically extract the pure fingerprint of the plastic.^43^

Non-negative matrix factorization (NMF) was conducted to extract plastic fingerprints from the recorded data set, forming the basis for constructing a multivariate standard addition curve (see Figure 1d). NMF models the recorded mass spectrum as a linear combination of pure variables^43^

2 $$r_{i,j}=\sum_{n=1}^{N}c_{i,n}s_{n,j}+e_{i,j}$$

where *r*_*i,j*_ is the recorded signal for the *i*th sample at the *j*th variable (i.e., *m*/*z* **),*e***_***ij***_ is the noise contribution that is minimized by the NMF algorithm, *N* denotes the different factors, *c*_*i,n*_ represents the factor concentration per subsample, and *s*_*n,j*_ is the factor source or fingerprint, i.e., the relative contribution of the different *m*/*z* to a factor. Note that *N* is 2 for our method in the ideal situation, namely, the sample matrix that is the same for all subsamples, and the PS fingerprint that varies due to the standard additions. In other words, each of the mass spectra measured by TD-PTR-MS (and integrated as described above) can be approximated by a linear combination of 2 factors. Each chemical entity can be represented as the multiplication of the factors ***c***_***i***, ***n***_ and ***s***_*n*,*j*_, which represent the concentration and fingerprint of the factor, respectively.

For matrix notation in linear algebra, we can rewrite eq 2 as

3 $$R_{I,J}=C_{I,n}S_{n,J}+E_{I,J}$$

The data matrix **R**_*I,J*_ with dimensions *I* (number of subsamples/measurements) by *J* (number of columns/ions) is the measured data. Factor concentrations and fingerprints/sources are described by the factor matrices ***C***_***I***, ***n***_ of dimensions *I* × *N* and **S**_**n**,**J**_ of dimensions *J* × *N*, respectively. **E**_**I**,**J**_ is the residuals/error matrix of dimensions *I* × *J* and stands for the variation unexplained by the bilinear model, where each column of **C**_**I**,**n**_ and **S**_**J**,**n**_ contains the concentration and mass profile (fingerprint) of a chemical entity, as **R**_**I,J**_ is the recorded mass data. The PS NPs are a source of signal in the measurement, and decomposition of the recorded data using eq 3 gives us the pure concentration of PS NPs per sample in ***C***_***I***, ***n***_ and the PS mass fingerprint in **S**_**J**,**n**_ (see Figure 1c).

There are several algorithms to decompose a measured data set into factors of concentration and fingerprint^42^ based on eq 3. Non-negative matrix factorization (NMF) with multiplicative updating is one of the widely employed algorithms.^44,45^ Given a matrix, ***R***_*I*×*J*_, it can be decomposed into the product of matrices ***C***_***I*** ×*n*_ and ***S***_*J*×*n*_ with an intrinsic non-negativity property. To formalize this, NMF optimizes the following cost function:

4 $$\min\left(C_{I\times n}\geq 0,S_{J\times n}\geq 0\right)∥R_{I\times J}-C_{I\times n}S_{n\times J}∥\_F$$

The algorithm tries to generate chemically meaningful factors (***C***_***I*** × ***n***_ and ***S***_***J*** × ***n***_), minimizing the cost function defined as the Frobenius norm || ||_F_ of the residual. The residual is the part of the data unexplained by the model, which is the difference between recorded measurements (***R***_*I*×*J*_) and reconstructed data (***C***_***I*** × ***n***_ **S**_***n*** × ***J***_).

Starting from rational initialization for ***C***_***I*** × ***n***_ and **S**_**J,n**_, the following updating steps are iteratively implemented until algorithm convergence.

5 $$S_{n\times J}\leftarrow S_{n\times J}\frac{\left(C_{n\times I}R_{I\times J}\right)}{\left({C_{n\times I}C}_{I\times n}S_{n\times J}\right)}$$

6 $$C_{I\times n}\leftarrow C_{I\times n}\frac{\left(R_{I\times J}S_{n\times J}\right)}{\left(C_{I\times n}S_{J\times n}S_{n\times J}\right)}$$

These element-wise multiplications preserve the non-negativity of component matrices, ***C***_***I*** × ***n***_ and ***S***_***J*** × ***n***_, where ***R***_*I,J*_ is element-wise non-negative.^46^ The signal in mass spectrometry is inherently noncontinuous (sparse), which means that only a limited number of *m*/*z* channels contain signals, while the rest can be forced to zero.^47,48^ This information can be implemented during curve resolution as a constraint, which helps in the accurate recovery of the pure signals possible to a unique solution.^48^ For sparse non-negative decomposition, a modified version of NMF under l0-norm minimization with a reliable and fast convergence is conducted.^47^

For all of the decomposition algorithms, the convergence is set as a maximum of 2000 iterations or when the difference between consecutive convergence steps is below 10^–6^. Finally, the convergence can be evaluated by means of the percentage of lack of fit (LOF):

7 $$LOF=100\times \sqrt{\frac{\sum_{ij}^{IJ}\left(r_{i,j}-{\mathrm{r̂}}_{i,j}\right)}{\sum_{ij}^{IJ}{\left(r_{i,j}\right)}^{2}}}$$

where *r*_*i*,*j*_ and ${\mathrm{r̂}}_{i,j}$ are the elements of the experimental and reproduced matrix based on eq 3, respectively. The LOF indicates the percentages of the measured data set not explained by the bilinear model dedicated to the contribution of noise and drift. The constraints imposed during NMF are non-negativity on both concentration and mass spectra and sparsity of mass channels.^43^ Despite spectroscopic signals (i.e., absorption spectra), mass signals have a noncontinuous trend (sparse). So, the sparsity constraint helps the NMF to recover meaningful mass spectra by forcing *m*/*z* channels to zero. A full version of the MATLAB pseudocode for this study is included in the Supporting Information.

## 3 Results and Discussion

### 3.1 Synthetic Samples

The applicability of the proposed multivariate standard addition method assisted with NMF on TD-PTR-MS was tested on synthetic samples to confirm the accuracy of the PS NP detection. Figure 2 panel a illustrates the MSA plots for the synthetic samples. The MSA plots show the added PS concentration vs the extracted NMF factor. The calculated concentration of PS NPs for nonspiked and synthetic samples was 0.087 ± 0.18 and 40 ± 0.66 ng/mL, respectively, showing no statistically significant difference from the expected values (0 and 40 ng/mL). The 95% confidence limit for each sample is calculated based on $\mathrm{X̅}\pm \frac{tS}{\sqrt{3}}$ , *t*_(0.975,2,two‑sided)_, where *X̅* and *s* are the mean and standard deviation of the three replicates, respectively.

**Figure 2:**
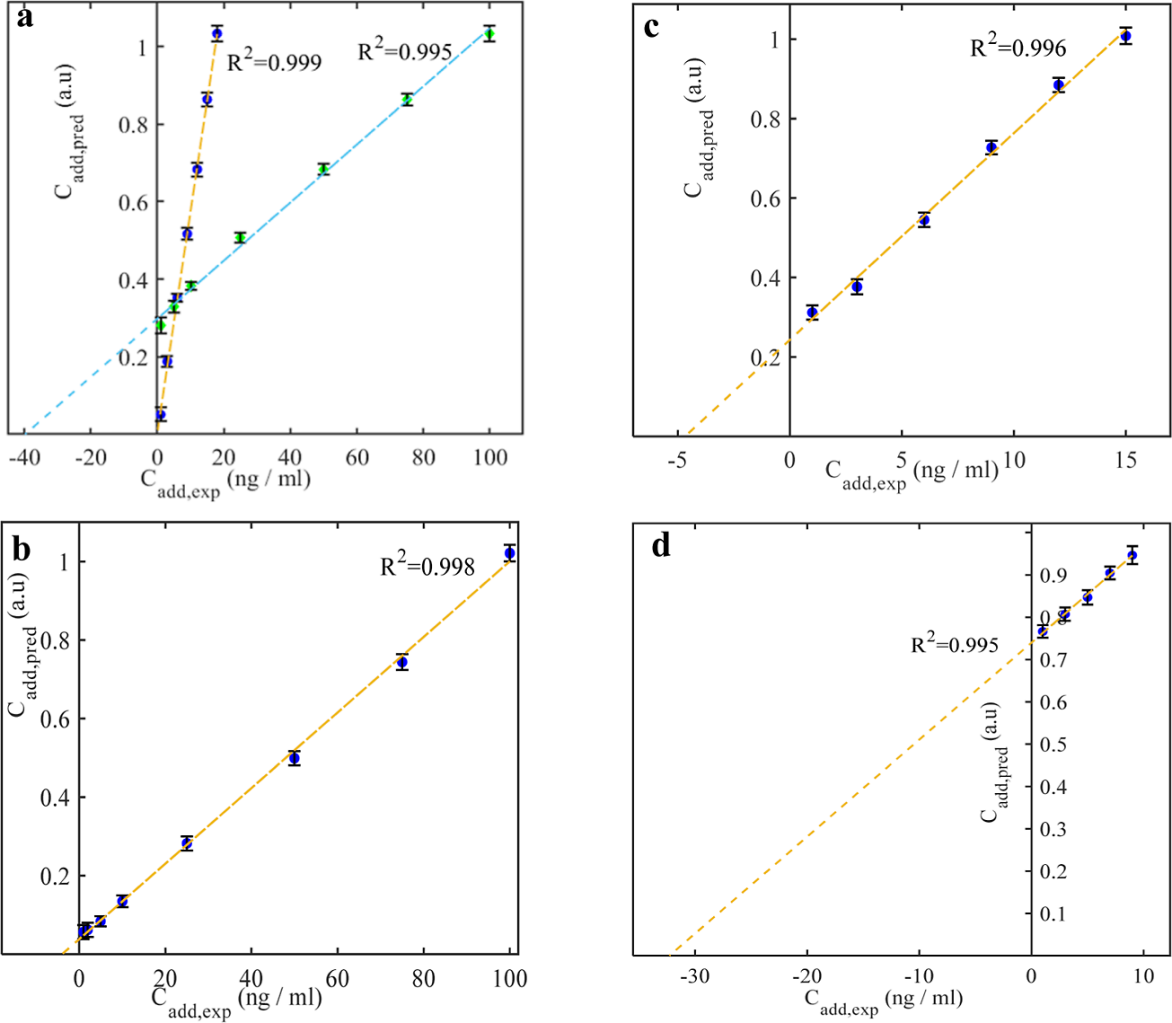
Multivariate standard addition plot for (a) synthetic samples with 0 (blue points) and 40 nanograms of spiked PS (green points), (b) tap water, (c) water from the Waal River, and (d) sand from the park. The MSA plots show the added polystyrene nanoplastic vs the predicted PS concentration from NMF. The *x*-intercept value represents the PS NP content in 1 mL of the analyzed sample. A factor of 4 can be applied to sand samples to convert to units of nanogram PS per gram of sand, as they undergo dilution during the sample preparation step. It should be noted that the first point on the curves is the first standard addition sample.

In the synthetic sample spiked with 40 ng/mL of PS NPs, the recovered value using a semiquantitative approach^11,15,32^ was 22 ± 12 ng/mL, substantially lower than the true value. Based on the results obtained from synthetic samples, the accuracy of the proposed NMF-MSA method is confirmed by retrieving the correct estimation of (non)-spiked samples. The results of the quantitative analysis of PS NPs based on our proposed framework and additionally on a semiquantitative approach, eq 1, are summarized in Table 1. The limit of detection (LOD) for each sample was calculated based on the procedure elaborated in Supporting Information. After calculating the pure contribution of the PS NPs, a standard calibration curve will be drawn (see Figure S1) for LOD calculation.

**Table 1:** A Summary of Quantification of Polystyrene in Different Samples Based on TD-PTR-MS^b^ ^,^ ^d^

samples specification	sample type	polystyrene MSA (this study)^a^	LOD	polystyrene semi-quantitative analysis^a^ Materić et al. refs 11 and 33
analytical blank (0 ng/mL)	HPLC water	0.087 ± 0.18 ng/mL	0.154 ng/mL	no match
spiked blank (40 ng/mL)	HPLC water	40 ± 0.66 ng/mL	0.16 ng/mL	22 ± 12 ng/mL
tap water—lab Utrecht^c^	tap water	3.9 ± 0.88 ng/mL	0.165 ng/mL	no Match
sandy park—Nijmegen	sand	129 ± 1.1 ng/g	0.173 ng/mL	67 ± 12 ng/g
Waal river—Nijmegen	water	4.7 ± 0.65 ng/mL	0.175 ng/mL	1.4 ± 0.80 ng/mL
Waal river—Nijmegen	sand	39 ± 0.70 ng/g	0.187 ng/mL	8.2 ± 10 ng/g

a The confidence interval for each item is calculated based on three replicates, $\mathrm{X̅}\pm \frac{tS}{\sqrt{3}}$ , *t*_(0.975,2,two‑sided)_.

b g, ng, and mL stand for gram, nanogram, and milliliter, respectively.

c Tap water from the laboratory at the Institute for Marine and Atmospheric research Utrecht (IMAU), located at Utrecht University, was used in this study.

d Where *X̅* and *s* are the mean and standard deviation of the three replicates, respectively. It should be noted that the sand samples were washed with HPLC water at a ratio of 1:4, while the results from the multivariate standard addition curves are for 1 mL of the extracted sample. Hence, a dilution factor of 4 was taken into account when reporting the total concentration of PS NPs.

### 3.2 Environmental Samples

Environmental samples comprised a more complex matrix when compared with synthetic ones. Assessing these samples involved determining the PS NPs in tap and river water as well as sand from a riverbank and a playground. The tap water and river water, respectively, contained 3.9 ± 0.88 and 4.7 ± 0.65 ng/mL of polystyrene NPs (see Figure 2, panels b and c). The PS NP contents of sand samples were 129 ± 1.1 and 39 ± 0.70 ng/g for the playground and Waal River shore, respectively (Figure 2).

We attribute the higher NP contents of sand samples to the nonspecific adsorption of nanoparticles on the sands.^49^ The MSA plots for the sand samples from the Waal River and playground are illustrated in Figures 1d and 2d. Furthermore, the high polystyrene content of the playground sand (compared to the river sands) may be due to air transportation or due to the built-in plastic structures in the park;^50^ i.e., abrasion of plastic from toys (e.g., sand molds) or mounted structures (e.g., rocking horses) increases the plastic budget of the playground. Additionally, the presence of PS nanoparticles in the river Waal matches the repeated detection of polystyrene microbeads in the lower river Rhine,^51^ i.e., upstream of our sampling location. In short, our results (Table 1) confirm the applicability of the proposed multivariate standard addition method assisted by NMF for samples with different matrices.

To minimize contamination during laboratory procedures, a robust and controlled workflow was implemented throughout all of the analytical steps. All sample preparation was carried out under a clean (HEPA + charcoal filter) laminar flow hood using prebaked, contamination-free vials to minimize background contamination. To further ensure clean conditions during thermal desorption, a constant stream of VOC-free zero air was used to carry the desorbed fragments into the PTR-ToF-MS instrument, preventing airborne contaminants from entering the system. Additionally, both Milli-Q and HPLC-grade water were routinely analyzed, and no detectable polystyrene nanoparticle contamination was found in either, confirming the absence of background interference from the water sources. For environmental samples, a field blank was also analyzed to account for potential contamination during sampling. Additionally, several process blanks were included, consisting of the PTFE filter, syringe, and pipet blanks, prepared by pouring ultrapure water through the entire sample preparation setup. No detectable polystyrene nanoparticles were found in either the field or the process blanks.

### 3.3 Comparison of MSA-NMF and the Semiquantitative Approach for PSNP Analysis Using TD-PTR-MS

Table 1 presents a comparison of polystyrene (PS) nanoparticle content in the analyzed samples, as determined using the MSA-NMF workflow proposed in this study and a semiquantitative approach based on the fingerprinting algorithm introduced by Materić et al.^33^ Generally, the semiquantitative method consistently underestimates PS nanoparticle concentrations by approximately a factor of 0.5 compared to values obtained using MSA-NMF. It was also unable to retrieve the PS fingerprint in samples with low PS NP content, such as tap water, further limiting its applicability in low-concentration matrices. This discrepancy can be attributed to limitations in thermal desorption efficiency and the presence of neutral fragments.^11,15,33^

The use of multiple spikings and knowledge of the exact concentration of added PS nanoparticles also contribute to the higher precision of the MSA-NMF method. In contrast, the semiquantitative approach exhibits greater uncertainty, particularly regarding confidence limits (see Table 1). A key advantage of the MSA-NMF approach is that it does not require an analytical blank, making it a blank-free method. Hence, PS nanoparticles are directly added to the test samples. Furthermore, the MSA-NMF workflow is fully quantitative, meaning the reported values have direct analytical significance, unlike the semiquantitative method.

## 4 Outlook

Nanoplastic quantification is complicated due to their limited abundance and the complexity of samples. Despite the development of several spectroscopic/spectrometric methods for nanoparticle quantification, various important issues need to be addressed. This study primarily focuses on method development. A multivariate version of the standard addition method assisted by signal extraction was suggested for the determination of NPs (here, polystyrene) in complex environmental matrices through the application of TD-PTR-MS.

The employed analytical method based on TD-PTR-MS can handle samples in different physical phases including liquid/solid/gas. The proposed method can be applied to a large variety of samples, such as seawater, sediments, and likely filtered air samples. It needs to be noted, however, that our results do not account for the retention of PS NPs by the used PTFE filter. In fact, Albignac et al.^52^ indicated that nearly all types of membranes exhibit retention effects on polymeric nanoplastics. It thus seems not likely that our measurements underestimate the actual levels of PS NPs.

Depending on the availability of standard plastic material, our workflow can be applicable to the detection and quantification of other polymers, too. The primary outputs of this approach encompass vital information, including nanoplastic-type detection by clustering the MS channels and full quantification. This characterization of NPs plays a pivotal role in accurately assessing the current state of nanoplastic pollution in different ecosystems. By utilizing this comprehensive approach, we aim to significantly enhance our understanding of nanoplastic distribution and abundance in complex environmental samples, ultimately contributing to informed environmental management and conservation efforts.

## Supplementary Material



## Notes

MATLAB pseudocode for TD-PTR-MS data analysis; details of LOD calculations; and justification for the thermal desorption program including thermogram and TGA curve (PDF)
