# Supplementary material for: Detecting Polystyrene Nanoparticles in Environmental Samples: A Comprehensive Quantitative Approach Based on TD-PTR-MS and Multivariate Standard Addition
Source: ACS ES T Water. 2025 Aug 1;5(9):5037–44. doi: 10.1021/acsestwater.5c00054 (PMC12439299; doi:10.1021/acsestwater.5c00054)
Supplement: Supplementary file 1 [file ew5c00054_si_001.pdf]

Detecting Polystyrene Nano-Particles in Environmental Samples: A Comprehensive Quantitative Approach Based on TD-PTR-MS and Multivariate Standard Addition

Nematollah Omidikia[1,2], Helge Niemann[1,3], Hanne Ødegaard Notø [2], Rupert Holzinger[2]

(1) Department of Marine Microbiology and Biogeochemistry (MMB), Royal Netherlands Institute of Sea Research (NIOZ), 1797 SZ 't Horntje, the Netherlands
(2) Institute for Marine and Atmospheric Research, IMAU, Utrecht University, Utrecht 3584 CC, The Netherlands.
(3) Department of Earth Sciences, Faculty of Geosciences, Utrecht University, Utrecht 3584 CB, The Netherlands.

Email: nemat.omidikia@nioz.nl

Supporting Information

1-MATLAB Code

The MATLAB pseudocode for the analysis of TD-PTR-MS data matrices coming from multivariate standard addition protocol is summarized as follows.

```
function NanoplasticDetection()   %%%% here NP is the Polystyrene
    % Step 1: Input Data
    processedData = load('path_to_raw_mass_spectra.mat'); % Load processed high-
resolution   mass spectra data
  C_added=load(standard addition concentrations)
   temperatureWindow = [150, 350]; % Set temperature integration window
    % Step 2: Average mass spectra for each sample
    averagedSpectra = AverageMassSpectra(processedData, temperatureWindow);
    % Step 3: Data Structuring
    dataMatrix = StructureDataMatrix(averagedSpectra); % Organize into matrix format
    % Step 4: Non-Negative Matrix Factorization (NMF) with L0 Regularization
    N = 2; % Define number of chemical components (2 is optimal)
    C = InitializeMatrix(size(dataMatrix, 1), N); % Concentration matrix
    S = InitializeMatrix(N, size(dataMatrix, 2)); % Spectral matrix
    maxIterations = 2000;  %%%% should be adjusted
    tolerance = 1e-6; %%%% should be adjusted
    sparsityLimit = 30; % Maximum non-zero elements allowed per column in S
    convergenceReached = false;
    iteration = 0;
    while ~convergenceReached && iteration < maxIterations
        iteration = iteration + 1;
        % Update S (spectral matrix) with L0 regularization
        S = UpdateSpectralMatrixWithL0(C, dataMatrix, sparsityLimit);
        % Update C (concentration matrix)
```

```
        C = UpdateConcentrationMatrix(S, dataMatrix);
        % Calculate residual and check for convergence
        residual = CalculateResidual(filteredData, C, S);
        if CheckConvergence(previousResidual, residual, tolerance)
            convergenceReached = true;
        end
        previousResidual = residual; % Store current residual for next iteration
    end
    % Step 5: Output generation
    OutputResults(C, S, residual);
    %Step 6: check the correlation of S rows with nanoplastic fingerprint in bank
Index_PS=matching_signature(S,NP_bank)
    % Step 7: drawing classical standard addition plot
Plot (C_added, C(index_PS)
    %Step 8: calculate the NP concentration
C_test_NP=x_intercept(C_added,C(index_PS)
    % Step 9: Optional Visualization
    PlotConcentrationProfiles(C);
    Plotpuresignature(S(index_PS));
end
% sub-functions (to be implemented as per your requirements)
function averagedSpectra = AverageMassSpectra(processedData, temperatureWindow)
averagedSpectra =sum(processedData(temperatureWindow(1):
temperatureWindow(2),:)
end
function dataMatrix = StructureDataMatrix(averagedSpectra);
    for i=1:N ; % N is number of standard addition samples
```

```
dataMatrix (i,:) = averagedSpectra (n);
    end
end
function C = InitializeMatrix(rows, cols)
    % Initialize matrix with non-negative values
    C = abs(rand(rows, 2));
end
function S = InitializeMatrix(2, cols)
    % Initialize matrix with non-negative values
    S = abs(rand(2, cols));
end
function S = UpdateSpectralMatrixWithL0(C, dataMatrix, sparsityLimit)
    % Implement the update for spectral matrix S with L0 regularization
    S = C' * dataMatrix; % Initial update (just as an example)
    % Apply L0 constraint by zeroing out entries exceeding sparsity limit
    for j = 1:size(S, 2)
        [~, idx] = sort(S(:, j), 'descend'); % Sort indices by value
        % Set to zero all but the top 'sparsityLimit' elements
        S(idx(sparsityLimit+1:end), j) = 0;
    end
    % Ensure non-negativity
    S = max(S, 0);
end

function C = UpdateConcentrationMatrix(S, dataMatrix)
    % Implement the update for concentration matrix C
    C = dataMatrix * S' / (S * S');
```

```
end
function residual = CalculateResidual(dataMatrix, C, S)
    % Calculate the residual using Frobenius norm
    R_hat = C * S; % Reconstructed data
    residual = norm(dataMatrix - R_hat, 'fro'); % Frobenius norm
end
function converged = CheckConvergence(previousResidual, currentResidual, tolerance)
    % Check for convergence based on residual
    converged = abs(previousResidual - currentResidual) < tolerance;
end
function OutputResults(C, S, residual)
    % Output the results, e.g., save to file or display
    disp('Concentration Matrix (C):');
    disp(C);
    disp('Spectral Matrix (S):');
    disp(S);
    disp(['Residual: ', num2str(residual)]);
end
function Index_PS=matching_signature(S,NP_bank)
correlation=corr(S,NP_bank)
[Index_PS,corr_PS]=max(correlation)
end
function C_PS_test=x_intercept(C_added,C(index_PS))
[slope, intercept]=cfit(1:N, C(index_PS),’poly1’)
C_PS_tes= - (intercept/slope)
end
function PlotConcentrationProfiles(C)
```

```
    % Implement visualization of concentration profiles
end
function PlotReconstructedSpectra(C, S)
    % Implement visualization of reconstructed spectra
End
```

2. LOD calculations

The limit of detection for multivariate standard addition followed by Non-Negative Matrix Factorization is calculated as follows. First, the recorded raw data matrix is deconvoluted using

sparse non-negative matrix factorization. The recorded data matrix adheres to a straightforward bilinear model, as follows:

$$\boldsymbol{D}_{I,J} = \boldsymbol{C}_{I,N}\boldsymbol{S}_{N,J} + \boldsymbol{E}_{\boldsymbol{I,J}} \qquad \text{equation (S1)}$$

Here, $\boldsymbol{D}_{I,J}$ represents the data matrix, while $\boldsymbol{C}_{I,N}$ and $\boldsymbol{S}_{N,J}$ are the concentration and spectral matrices recovered from the model, and $\boldsymbol{E}_{\boldsymbol{I,J}}$ denotes the unmodeled portion of the dataset. After the model converges, the concentration and spectral profiles for polystyrene can be identified within the component matrices. We then denote these as $\boldsymbol{c^{PS}}_{I,1}$ and $\boldsymbol{s^{PS}}_{J,1}$, representing the pure concentration and mass spectrum of polystyrene extracted from the model. The overall contribution of polystyrene nanoparticles, $\boldsymbol{D^{PS}_{I,J}}$, in the raw data $\boldsymbol{D}_{I,J}$ can be calculated as:

$$\boldsymbol{D^{PS}_{I,J}} = \boldsymbol{c^{PS}}_{I,1}\boldsymbol{s^{PS}}_{1,J} \qquad \text{equation (S2)}$$

In this manner, the pure contribution of polystyrene nanoparticles in each row of the dataset (where each row corresponds to a standard addition sample) is calculated. This pure contribution can also be regarded as the net analyte signal of the PS nanoparticles, which can then be used for LOD calculations. The Frobenius norm of each row (i=1,…,I) is formulated as follows: $\|\boldsymbol{s^{PS}}_{i,J}\|_F = \sqrt{\sum_{j=1}^{J} |\boldsymbol{s^{PS}}_{i,j}|^2}$ equation (S3)

A calibration curve can then be created, plotting the nominal concentration of PS-NPs on the x-axis and $\|\boldsymbol{s^{PS}}_{i,J}\|_F$ as y-axis, with m representing the slope. Then the LOD can be calculated as:

$$LOD = \frac{3\sigma_b}{m} \qquad \text{equation (S4)}$$

In Equation (S4), σ_b represents the standard deviation of the blank signal retrieved from NMF. For the sake of exemplification, Figure S1 shows the standard calibration curve for the first sample in Table 1 (analytical blank without spike).

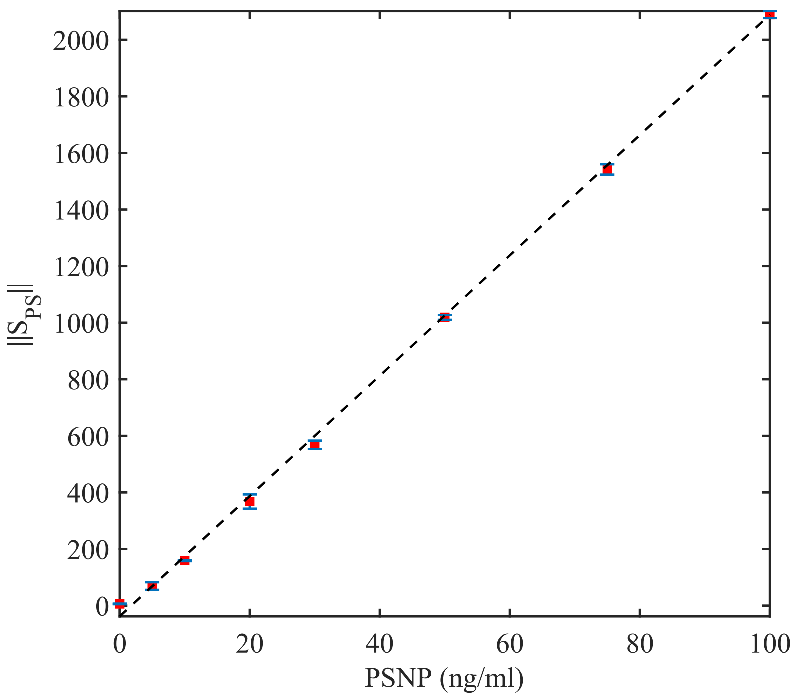

Figure S1. Standard calibration curve for the analytical blank without a spike (refer to the first row in Table 1). The x-axis represents the concentration of PS NPs, and the y-axis shows the pure contribution of PS NPs in each standard addition subsample, calculated using Equation S4.

3-Thermal Desorption (TD) program Justification

To justify the thermal desorption (TD) program—where the temperature was ramped from 35 °C to 350 °C at a rate of 40 °C/min and then held at 350 °C for 5 minutes—the thermogram of the styrene ion (m/z 105.07), a key pyrolysis product of polystyrene, was drawn and is presented as Figure S2. The PSNP signal shows a pronounced peak during the heating phase and declines substantially within the 5-minute period at 350 °C. This indicates that the majority of polystyrene nanoparticle degradation occurs within this time frame. Therefore, holding the temperature at 350 °C for 5 minutes is sufficient for complete thermal decomposition of the PSNPs.

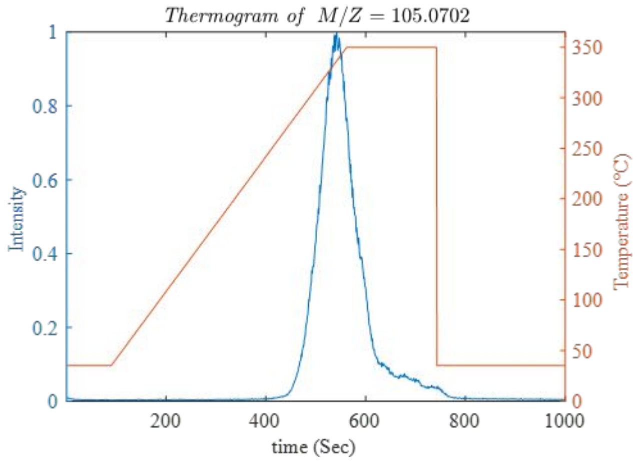

Figure S2. Thermogram of Styrene ion for a solution containing 60 ng/ml PSNPs.

As supporting evidence, the TGA curve (PerkinElmer TGA 8000 instrument) obtained from a 60 µL aliquot of a 5% w/v PSNP solution (300 nm diameter), corresponding to approximately 3 mg of PS was recorded (see Figure S3). The TGA curve shows that once the temperature reaches 350 °C, it takes approximately 5–7 minutes to completely decompose the polystyrene content. This observation supports the adequacy of the thermal desorption program used for complete degradation of PSNPs at 350 °C.

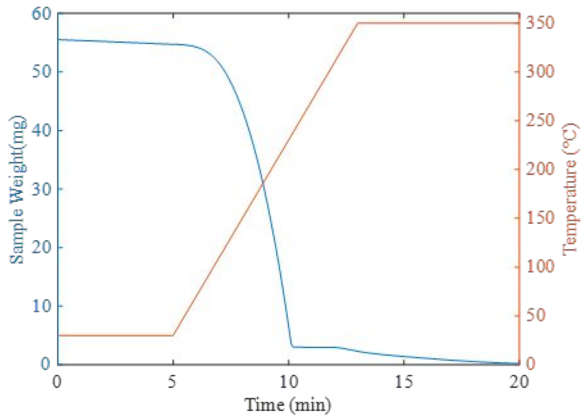

Figure S3. TGA curve for 60 µL aliquot of a 5% w/v PSNP solution.
